# Tyrosine nitration of cytosolic peroxidase is probably triggered as a long distance signaling response in sunflower seedling cotyledons subjected to salt stress

**DOI:** 10.1371/journal.pone.0197132

**Published:** 2018-05-16

**Authors:** Prachi Jain, Satish C. Bhatla

**Affiliations:** Laboratory of Plant Physiology and Biochemistry, Department of Botany, University of Delhi, Delhi, India; Institute of Genetics and Developmental Biology Chinese Academy of Sciences, CHINA

## Abstract

Present work focuses on tissue and concentration-dependent effect of nitric oxide (NO) on the modulation of cytosolic peroxidase (POD; EC 1.11.1.7) activity in 2-day old etiolated sunflower (*Helianthus annuus* L.) seedlings. Exogenously supplied NO (in the form of sodium nitroprusside [SNP] or diethylenetriamine NONOate [DETA]; 125 to 500 μM) results in noteworthy enhancement in seedling growth in a concentration dependent manner irrespective of salt-stress and differentially affects POD activity in 2-day old seedling cotyledons. Elevated NO availability leads to an increase in the specific activity of POD in a concentration-dependent manner within 48 hrs as a rapid signaling response. Purification of POD protein using immunoprecipitation technique has shown that cotyledons derived from salt stressed seedlings exhibit a higher extent of tyrosine nitration of POD as compared to the control seedlings. Out of the four tyrosine residues found in the amino acid sequence of POD, the one at position 100 has been predicted to undergo nitration. Thus, a probable NO-POD crosstalk is evident in sunflower seedling cotyledons accompanying salt stress.

## Introduction

**Fig 1:**
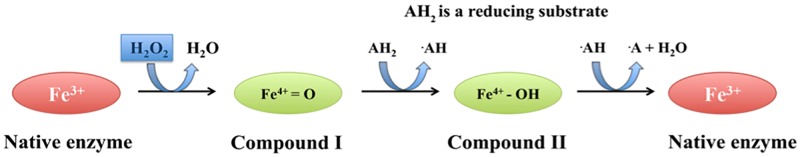
The reaction mechanism of plant peroxidases.

Salt stress is known to cause a reduction in seed germination response, disturbance in ion uptake, alterations in gaseous exchange, and destruction of the photosynthetic machinery, thereby causing decreased photosynthetic yield in plants [1]. Sunflower is a moderately salt-tolerant crop [2]. Different environmental factors, including salinity, greatly influence the growth of sunflower plants and yield of seeds [3]. The hyperionic and hyperosmotic effects of salinity experienced by plants leads to membrane disorganization, increase in the levels of reactive oxygen species (ROS), and metabolic toxicity [4]. Salt stress induces oxidative stress which results in generation of reactive oxygen species (ROS), such as hydroxyl radical (OH), superoxide anion (O_2_^.-^), singlet oxygen (^1^O_2_) and hydrogen peroxide (H_2_O_2_). ROS scavenging is one of the common defense responses against abiotic stresses [5]. Plants are equipped with an elaborate ROS scavenging network, composed of both non-enzymatic and enzymatic mechanisms, for regulating intracellular ROS levels [6]. The most common targets of antioxidant enzymes under salt stress are O_2_^.–^and H_2_O_2_. The role of H_2_O_2_-detoxifying enzymes is to impose a tight control on its cellular concentrations rather than to remove it completely [7]. Peroxidases (POD, EC 1.11.1.7) are heme-containing oxidoreductases which catalyze the reduction of hydrogen peroxide by oxidizing a wide variety of electron donor substrates, including phenols, aromatic amines, auxins, fatty acids, liginin precursors and secondary metabolites [8]. Several peroxidase isozymes, differing in their molecular mass as well as catalytic properties, occur in different plant species [9]. The diversity of processes catalyzed by PODs as well as the large number of genes encoding POD suggests a possible functional specialization for each isoform [10]. PODs occur in three forms in plants namely soluble, covalently bound and ionically bound. The soluble forms are largely found in the cell cytoplasm while the ionic and covalently bound forms are associated with cell wall and some cell organelles. PODs are also classified as acidic or basic according to their isoelectric points [11]. Most peroxidases follow the reaction scheme as shown in Fig 1.

The three-step reaction cycle catalyzed by PODs involves a two-electron oxidation of the ferric enzyme (resting state) by H_2_O_2_ to form Fe^4+^ = O (Compound I). This is followed by two subsequent reduction reactions in which many electron donor substrates, mainly of phenolic nature, get oxidized themselves leading to the formation of Fe^4+^-OH (compound II) and finally the enzyme is converted back to its ferric form [12]. The enzyme nomenclature according to International Union of Biochemistry and Molecular Biology has listed around 15 different types of peroxidases out of which 5 are found in plants. They are: catalases (EC 1.11.1.6), peroxidases (EC 1.11.1.7), also called as class III peroxidases or guaiacol peroxidases or secreted peroxidases, glutathione peroxidases (EC 1.11.1.9), ascorbate peroxidases (EC 1.11.1.11) and peroxiredoxins (EC 1.11.1.15). Generally, most plants exhibit enhanced peroxidase activity upon exposure to oxidative and abiotic stresses. The upregulation of POD activity in response to salinity has been observed in numerous plants [13].

Nitric oxide (NO) is a bioactive, gaseous free radical which plays critical roles as a diffusible intracellular signaling molecule. NO is known to regulate various physiological and developmental processes in plants, including seed germination [14], induction of lateral roots [15], flowering [16], adventitious root formation [17], and hormonal responses [18]. High reactivity of NO radical induces different post-translational modifications in a wide range of proteins, lipids and nucleic acids, thereby affecting their structure and functions [19]. NO reacts with thiol or heme-containing proteins, thus altering their functions and/or structure [20]. It may bind to Cys residues, iron or heme centres or tyrosine residues in proteins thereby altering their structure and function [19]. Most of the biological regulatory properties of NO are governed by its intrinsic instability, lipophilicity, and high affinity towards iron, thus making it a very good agent for both signal transduction and defense responses [6]. NO plays a protective role in plants by regulating the level and toxicity of ROS. There are possibly two mechanisms by which NO counteracts oxidative stress. First, NO directly scavenges ROS, such as O_2_·-, to form peroxynitrite (ONOO-). Secondly, NO might also function as a signaling molecule in the cascade of events thereby altering gene expression [21]. NO has been reported to exert protective effect in response to drought stress [22], osmotic stress [23], salt stress [24, 25, 26], high temperature stress [24], and heavy metal stress [21, 27, 28].

Present work focuses on NO-mediated modulation of class III peroxidases or secretory/cytosolic peroxidases in 2-day old sunflower seedling cotyledons. Although quite a few reports on NO actions in plants using various NO donors (SNP, SNAP, GSNO) are available, the effects of these compounds can, however, differ depending on the nature and concentration of the NO donor used. Moreover, saturating doses of NO provided to plants through exogenous application of NO donors, cannot always justify their actual physiological roles. Depleting the plant tissue of its endogenous NO by the application of NO quencher (commonly cPTIO) is another way to understand the physiological relevance of endogenous NO in various responses [29]. Moreover, both abiotic stress and NO can evoke differential responses in roots and in terms of long distance sensing of stress to aerial plant parts. Further, these differences vary according to concentration and nature of NO donor used. Recent investigations carried out in the author’s laboratory have shown the modulation of various antioxidative enzymes, such as glutathione reductase, superoxide dismutase and heme oxygenase by nitric oxide accompanying long distance sensing of salt stress in sunflower seedling cotyledons [30, 31, 20]. Keeping in mind a critical role of H_2_O_2_ accumulation as a component of ROS signaling in response to salt stress, present investigations provide new information on the regulatory role of cytosolic peroxidase (POD) in ameliorating salt stress through the modulation of enzyme activity and POD protein abundance. Furthermore, our findings provide new evidence for clear and differential impact of salt stress on NO-POD crosstalk in terms of abundance of enzyme, its activity and its modulation in terms of long distance sensing of salt stress. The work further provides evidence for concentration-dependence of the biochemical response due to NO through application of variable doses of two NO donors (SNP, DETA), NO scavenger (cPTIO) and putative inhibitor of arginine-dependent enzyme NO biosynthesis (aminoguanidine).

## Material and methods

### Plant growth and treatments

For germination, sunflower seeds (*Helianthus annuus* L. cv., KBSH 53) procured from University of Agricultural Sciences, Bangalore were washed with a liquid detergent and kept in running tap water for 1 hr. Seeds were sterilized using 0.005% mercuric chloride, washed and imbibed in distilled water for 2 hrs. Seeds were then spread on two layers of germination paper layered in plastic trays and irrigated with half-strength Hoagland nutrient medium. For providing salt stress conditions, Hoagland solution was supplemented with 120 mM NaCl based on earlier published work with regard to optimization of seedling growth under varying NaCl concentrations [25]. Seeds were germinated in dark at 25±2°C. In order to examine the effect of nitric oxide on peroxidase (POD) activity, Hoagland medium was supplemented with different concentrations (125, 250 and 500 μM) of sodium nitroprusside (SNP; NO donor), diethylenetriamine NONOate (DETA; NO donor), aminoguanidine (inhibitor of arginine-dependent NO biosynthesis) and 2- (4-Carboxyphenyl)-4,4,5,5-tetramethylimidazoline-1-oxyl-3-oxide (cPTIO; NO scavenger). The respective treatments were provided to seedlings in the nutrient medium, after the emergence of radical in the absence or presence of 120 mM NaCl. Cotyledons from 2d old sunflower seedlings showing uniform growth patterns were collected for various biochemical analyses and stored at -80°C until further use. It was decided to carry out all analyses on 2d old seedling cotyledons only, in order to focus on rapid signaling events taking place within 48 hrs of sensing of salt stress. For control SNP treatments, seedlings were also raised in the presence of exhausted SNP solutions (SNP exposed to light).

### Estimation of peroxidase (POD) activity

Peroxidase (POD) activity was estimated using the following protocol with minor modification [32]. Cotyledons, frozen with liquid nitrogen, were ground to fine powder using a pestle and mortar and dispersed in 50 mM sodium acetate buffer (pH 4.0) (1:2 w/v). The homogenates thus obtained were centrifuged at 10,000 g for 20 min at 4°C. Protein was quantified using Bradford assay [33] and 600 μL aliquot containing 25 μg protein was added to a 2.4 mL of substrate solution (0.6 mM o-dianisidine, 8.8 mM H_2_O_2_ in 50 mM sodium phosphate buffer, pH-6) to make the final volume to 3 mL. Change in absorbance was recorded at 460 nm for 5 min at an interval of 1 min each. Substrate solution without protein served as blank. Enzyme activity was expressed as specific activity of POD as moles.min^-1^.μg^-1^ protein. For zymographic detection of POD activity, protein extracted and quantified as described above was constituted in Laemelli buffer without SDS. Twenty-five microgram protein from each sample was loaded for one dimensional separation of polypeptides on a 12% vertical native polyacrylamide gel at 4°C (conditions: 75V for 20 minutes, 150 V for 2 h) using Miniprotean Tetra Cell (Biorad, USA). Gel with resolved bands was then incubated in 0.2 M sodium acetate buffer (pH 5.0) containing 1.3 mM benzidine and 1.3 mM H_2_O_2_ until the development of brown bands representing POD activity.

### Western blot detection of peroxidase (POD) isoforms

POD isoforms were detected by western blot according to the following protocol [9]. Briefly, cotyledons were ground to a fine powder with liquid nitrogen and homogenized in grinding medium containing 0.1 M Tris-HCl, 0.4 M sucrose, 10 mM KCl, 1 mM MgCl_2_, 1 mM EDTA and 1 mM PMSF (pH-7.5). The homogenates were centrifuged at 10,000 g for 20 min at 4°C to obtain total soluble protein (TSP). Twenty-five micrograms of each protein sample were loaded for one dimensional separation using 10% SDS vertical polyacrylamide gel in MiniProtean Tetra Cell (Biorad, USA) (conditions: 75V for 20 minutes, 150 V for 2 h). Gel with resolved bands was removed and washed in transfer buffer at 4°C for 15 min. Proteins were transferred to nitrocellulose (NC) membrane at a current of 400 mA for 1 h at 4°C. Subsequently the blot was incubated in blocking buffer [7% BSA, 0.2% Tween 20 in PBS, pH 7.4] for 2 hrs at room temperature, followed by overnight incubation at 4°C on an orbital shaker with anti-peroxidase antibody produced in rabbit (cat no. P7899 obtained from Sigma-Aldrich Chemicals Pvt. Ltd., USA) in a dilution of 1:2000 prepared in blocking buffer. Membrane was subsequently washed thrice in wash buffer (0.2% Tween-20 in PBS pH 7.4) for 5 min each and incubated on an orbital shaker in secondary antibody (anti- rabbit IgG conjugated to alkaline phosphatase antibody obtained from Sigma-Aldrich Chemicals Pvt. Ltd., USA) dispersed in 1:2500 ratio in wash buffer for 1 hr at room temperature. Finally, the membrane was washed thrice in wash buffer for 5 min each and developed using freshly prepared BCIP/NBT.

### Immunoprecipitation of peroxidase (POD) using Dynabeads Protein G and anti-POD antibody

**Fig 2:**
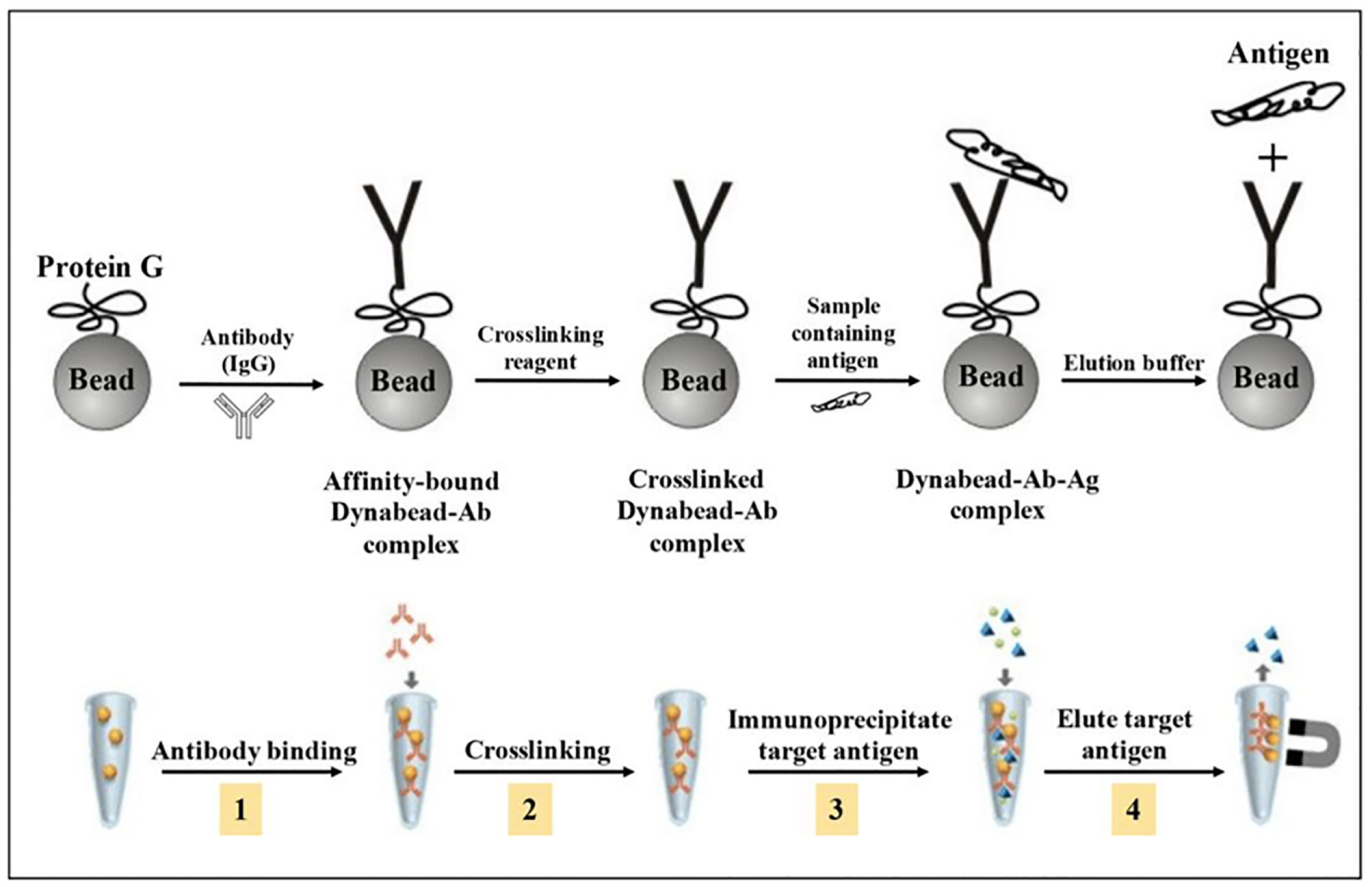
Schematic representation of immunoprecipitation protocol for protein purification.

POD was purified from seedling cotyledons using Dynabeads Protein G Immunoprecipiation kit (Invitrogen, USA), according to manufacturer’s instructions. The Dynabeads separated from the solution were incubated with anti-POD antibody (P7899, Sigma-Aldrich Chemicals Pvt. Ltd.) diluted in 200 μL antibody binding and washing buffer (provided in the kit) for 1 h at room temperature (RT) with constant rotation. After incubation, the tubes were placed on magnet and supernatant was removed. The Dynabeads-antibody (Ab) complex so obtained was further resuspended in 200 μL antibody binding and washing buffer to wash off unbound antibody. To prevent co-elution of antibody, the Dynabeads were crosslinked with anti-POD antibody using BS3 [bis(sulfosuccinimidyl)suberate] which is a crosslinking reagent. The Ab coupled-Dynabeads were washed twice in 200 μL of conjugation buffer [20 mM sodium phosphate buffer containing 0.15 M NaCl (pH 7.0)]. The beads were then resuspended in 250 μL of 5 mM BS3 solution and incubated with rotation at RT for 30 min. 12.5 μL of quenching buffer (1 M Tris-HCl, pH 7.5) was then added and the mixture was incubated again for 15 min at RT with rotation. The beads were washed thrice with washing buffer (provided in the kit), placed on the magnet and supernatant was discarded. The crosslinked Dyanbead-Ab complex was subsequently resuspended in 1000 μL of antigen (POD protein) containing sample (10,000g supernatant) and incubated for 2 h at RT under continuous rotation to allow antigen (Ag) to bind to Dynabeads-Ab complex. The tube was then placed on magnet and supernatant was removed. The Dynabead-Ab-Ag complex was washed thrice with 200 μL washing buffer and finally the Dynabead-Ab-Ag complex resuspended in 100 μL washing buffer was transferred to a fresh eppendorf tube to prevent co-elution of proteins bound to the wall of the tube. Finally, POD was eluted by gently resuspending the beads complexed with antigen and antibody in 20 μL of elution buffer (provided in the kit). The beads were subsequently incubated at room temperature for 5 min and then placed on the magnet. The supernatant (containing purified POD) was removed and stored for further analysis (Fig 2).

### Western blot detection of tyrosine nitration of peroxidase (POD) purified by immunoprecipitation

Aliquot containing purified POD obtained by immunoprecipitation was loaded for one dimensional separation on 10% SDS-PAGE using a Miniprotean Tetra Cell (Biorad, USA). Electrophoresis was performed at 75V for 0.5 h, 100V for 0.5 h and 150V for 2 hrs. After electrophoresis, the gel was removed from the cassette and washed in transfer buffer at 4°C for at least 15 min. Protein was transferred onto PVDF membrane at a current of 400 mA applied for 1 h. The membrane with transferred proteins was then incubated overnight at 4°C in blocking buffer and further with anti-3NT antibody (1:1000 diluted in blocking buffer) for 2 h at room temperature on an orbital shaker. Thereafter, the membrane was washed thrice in wash buffer for 5 min each and incubated in secondary antibody (anti-rabbit IgG conjugated to alkaline phosphatase, antibody) diluted 1:3000 in wash buffer for 1 h at room temperature. Finally, the membrane was washed thrice in wash buffer for 5 min each and developed using freshly prepared BCIP/NBT. Both, the primary and secondary antibodies were obtained from Sigma-Aldrich Pvt Ltd (USA).

### Statistical analysis

Statistical significance of treatment-induced changes in peroxidase (POD) activity were analyzed by one-way ANOVA using SPSS 22.0 in comparison with their respective controls. All experiments were performed at least thrice.

## Results

### Exogenously supplied NO positively modulates seedling growth and also significantly counters growth inhibition by NaCl stress

**Fig 3:**
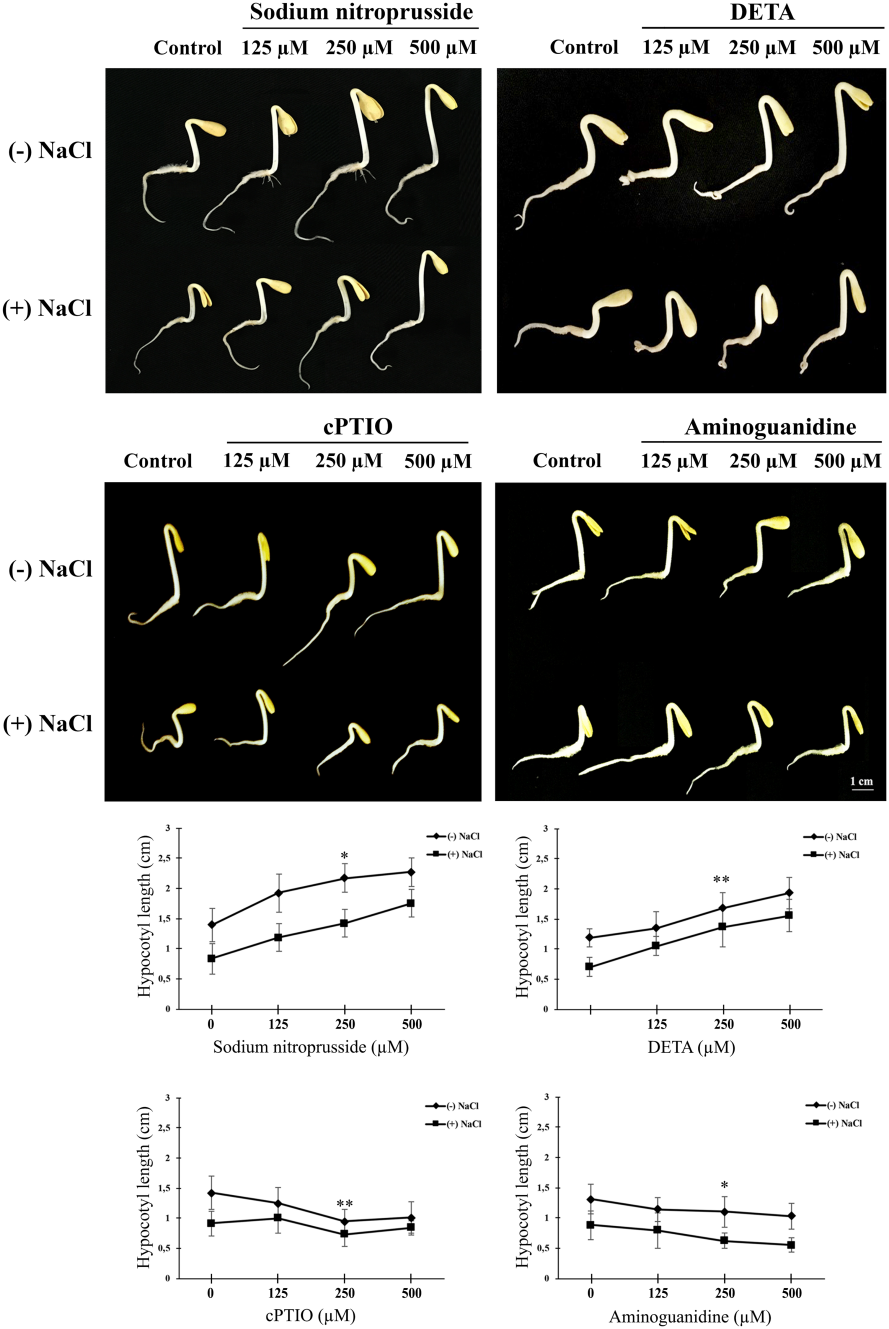
Changes in whole seedling growth and hypocotyl extension in response to varying concentrations (0 to 500 μM) of NO donors (SNP and DETA), an NO scavenger (cPTIO) and an inhibitor of arginine-dependent NO biosynthesis (aminoguanidine) in 2-day old sunflower seedlings grown in the absence or presence of 120 mM NaCl.

Exogenously supplied NO (in the form of SNP or DETA; 125 to 500 μM) resulted in noteworthy enhancement in seedling growth in a concentration dependent manner, both in control and salt-stressed seedlings (Fig 3). In order to further confirm the protective effect of NO on seedling growth observed in the present work, sunflower seedlings were also grown in Hoagland medium supplemented with different concentrations (125 to 500 μM) of cPTIO (NO scavenger) or aminoguanidine (inhibitor of putative NOS activity). Fresh weight data from a previous publication from author’s laboratory demonstrates that SNP leads to an increase in fresh weight of whole sunflower seedlings while application of aminoguanidine and cPTIO brings about a decrease in the fresh weight in a concentration dependent manner [34]. Exogenously supplied NO donors (SNP and DETA) resulted in significant enhancement of hypocotyl length. SNP and DETA led to 21.1% and 22.1% increase in hypocotyl length respectively, in the presence of salt stress (Table 1). However, aminoguanidine and cPTIO decreased the growth of hypocotyls (16% and 14% respectively) both in the absence or presence of 120 mM NaCl (Fig 3).

**Table 1:** Effect of NO on hypocotyl length of 2-day old sunflower seedlings.

Treatment	Concentration (μM)	Percent change in hypocotyl length (%)*	
		Control (-NaCl)	(+) NaCl
SNP	125	13.8% increase	14.21% increase
	250	15.6% increase	17.1% increase
	500	16.3% decrease	21.1% increase
DETA	125	13.4% increase	15% increase
	250	14.1% increase	19.4% increase
	500	16.2% increase	22.1% increase
cPTIO	125	11.5% decrease	10.9% increase
	250	16.9% decrease	12.5% decrease
	500	14.1% decrease	10.8% decrease
Aminoguanidine	125	11.5% decrease	11.1% decrease
	250	11.9% decrease	14.1% decrease
	500	12.7% decrease	16% decrease

* Percent change refers to change in hypocotyl length with respect to control (0 μM)

### Modulation of POD activity by NO in seedling cotyledons as a long-distance response

**Fig 4:**
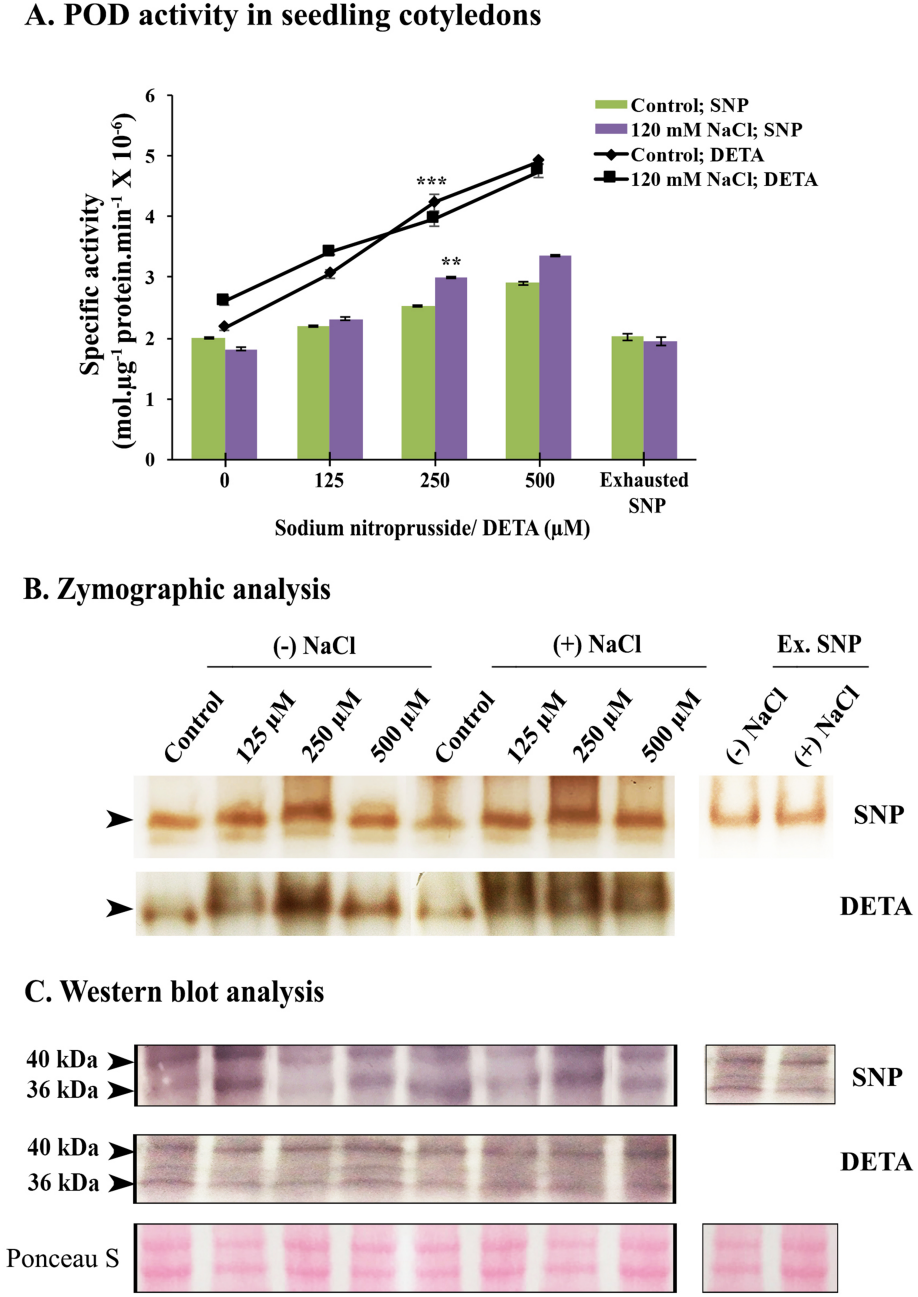
POD activity in cotyledons from 2d old sunflower seedling raised in the presence or absence of 120 mM NaCl and supplemented with different concentrations of sodium nitroprusside and DETA (125, 250 and 500 μM) in the Hoagland medium. (A) Spectrophotometric determination of specific activity of POD. Inset represents relative change in POD activity (% of respective control). Changes in enzyme activity were analyzed by one-way ANOVA using SPSS 22.0 and were found to be statistically significant (***p<0.001, **p<0.01) in comparison to control. Vertical bars on mean values in A represent SE (n = 3). (B) Zymographic detection of POD activity. (C) Western blot analysis of POD. (Ex. SNP- Exhausted 250 μM SNP).

Cotyledons derived from seedlings raised in variable concentrations (125–500 μM) of SNP or DETA exhibited concentration-dependent increase in POD activity as well as a clear difference in the sensitivity of the tissue to the available elevated concentrations of nitric oxide in NaCl stressed seedlings than those derived from control (-NaCl) conditions (Fig 4). The increase in POD activity in the presence of DETA (22.6% increase with respect to control) is, however, much more significant in control seedlings (- NaCl condition). The use of two donors (SNP and DETA) in the present work revealed similar changes in POD activity suggesting that the observed increase in POD activity is mainly due to additional availability of nitric oxide to the seedling cotyledons irrespective of salt stress. Exhausted SNP treatment (250 μM) however did not bring about any significant changes in enzyme activity in sunflower seedling cotyledons. These observations are consistent with the activity demonstrated zymographically as well. Zymographic analysis of the 10,000g supernatant from the cotyledon homogenates clearly showed the enhancing effect of SNP or DETA as NO donors on POD activity. The Western blot analysis of POD isoform in seedling cotyledons revealed the expression of two bands of 40 and 36 kDa. It is thus evident that elevating NO availability to the cotyledons of sunflower seedlings through an external source (SNP/DETA) leads to an increase in the specific activity of POD in a concentration-dependent manner within 48 hrs as a rapid signaling response.

**Fig 5:**
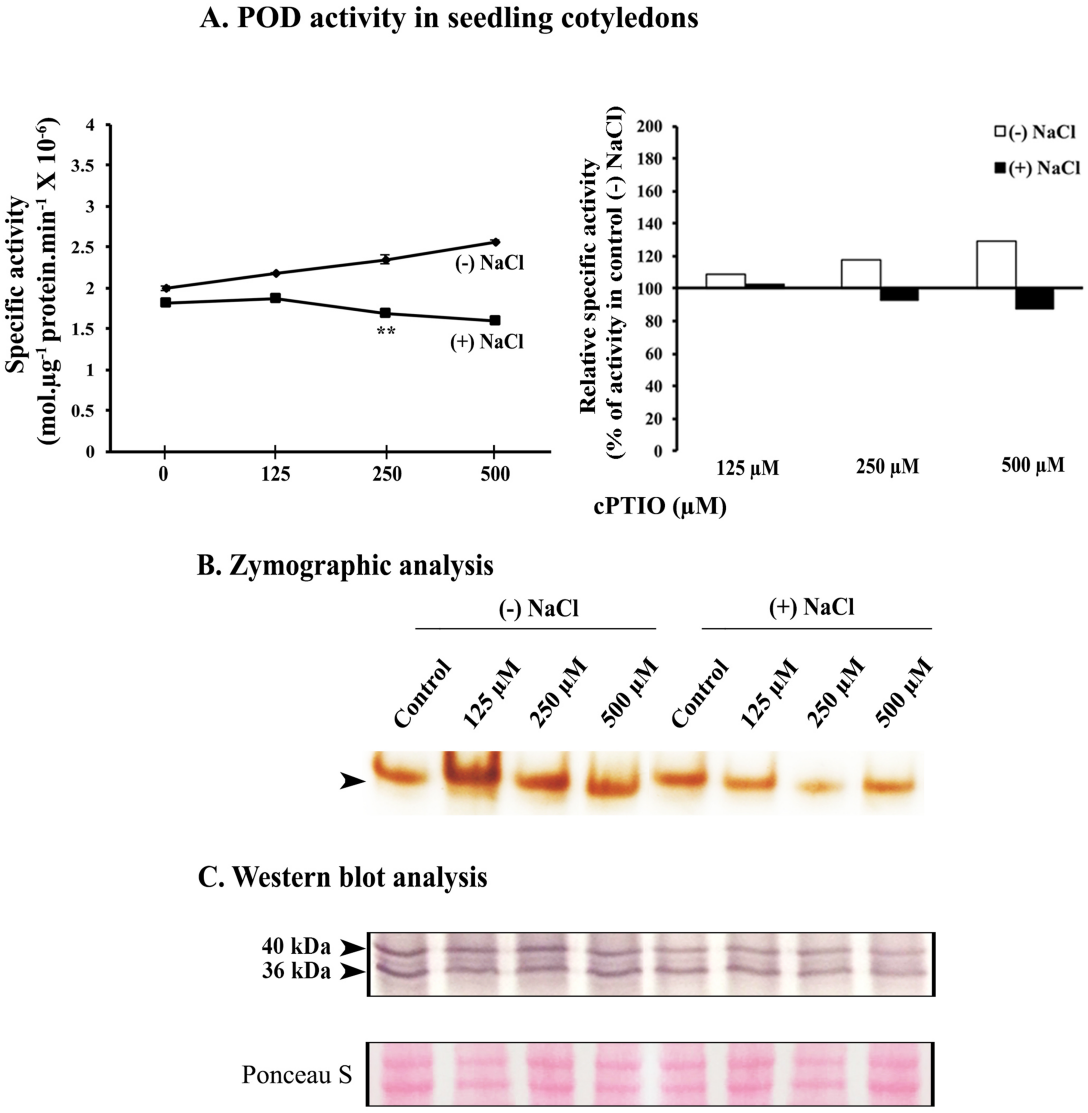
Effect of cPTIO (NO scavenger) on POD activity in cotyledons. 2d old sunflower seedlings were raised in the presence or absence of 120 mM NaCl and Hoagland medium was also supplemented with different concentrations of cPTIO (125, 250 and 500 μM). (A) Estimation of specific activity of POD. Inset represents relative change in POD activity (% of respective control). Changes in enzyme activity were analyzed by one-way ANOVA using SPSS 22.0 and were found to be statistically significant (**p<0.01) in comparison to control. Vertical bars on mean values in A represent SE (n = 3). (B) Zymographic detection of POD activity. (C) Western blot analysis of POD.

cPTIO, a well-known quencher of endogenous NO, significantly lowered POD activity at 250 μM (10.8% with respect to control) in the salt stress conditions (Fig 5). Furthermore, Western blot analysis of POD in homogenates derived from seedling cotyledons raised in various concentrations of cPTIO did not exhibit any major variation in control seedlings but it lowered down amount of POD protein in a concentration-dependent manner in seedling cotyledons derived from salt stressed seedlings (Fig 5). Zymographic analysis revealed similar trend as observed from the spectrophotometric data.

**Fig 6:**
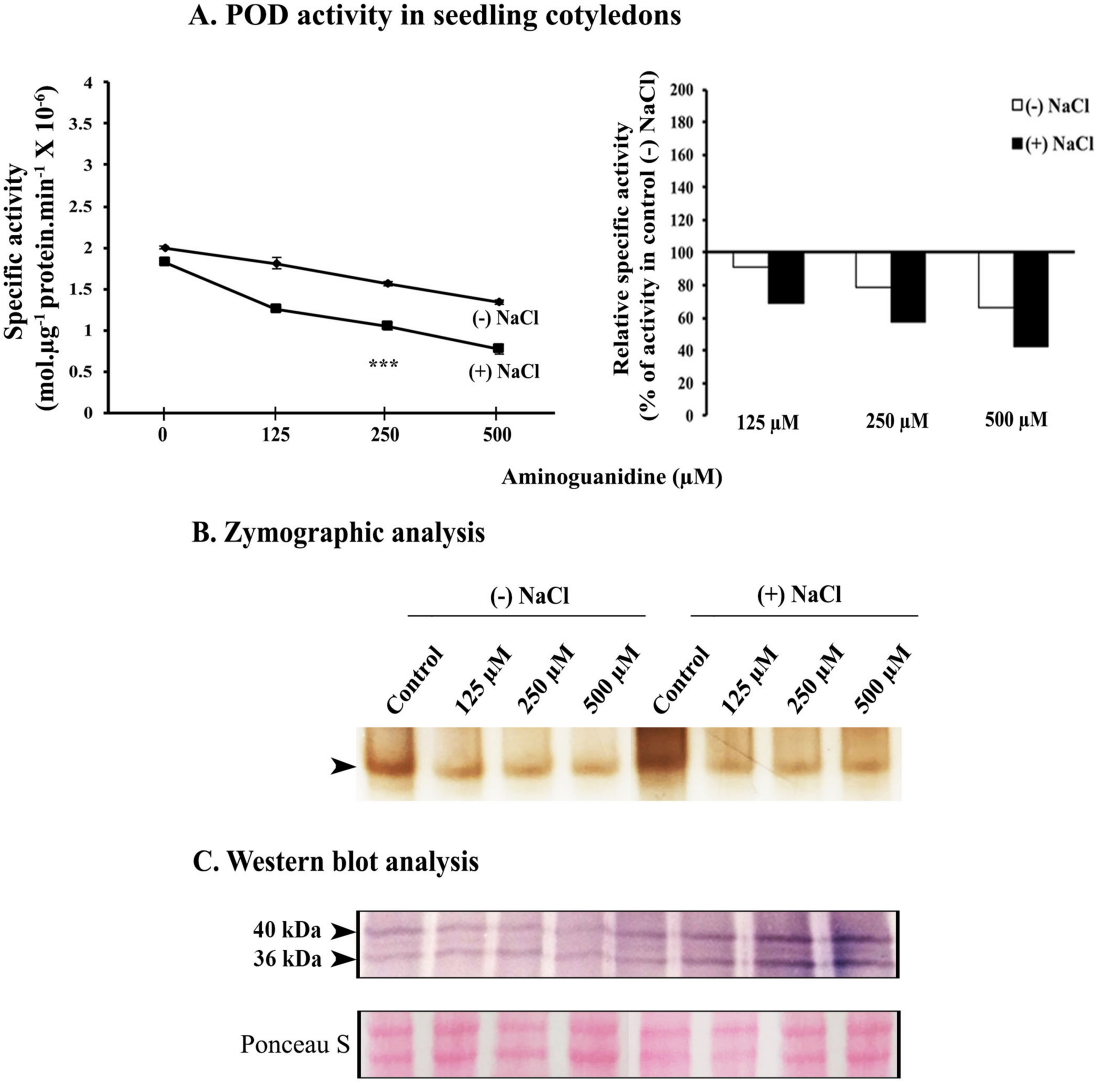
Effect of aminoguanidine (NOS inhibitor) on POD activity in cotyledons from 2d old sunflower seedlings raised in the absence or presence of 120 mM NaCl and supplemented with different concentrations of aminoguanidine (125, 250 and 500 μM). (A) Estimation of specific activity of POD. Inset represents relative change in POD activity (% of respective control). Changes in enzyme activity were analyzed by one-way ANOVA using SPSS 22.0 and were found to be statistically significant (***p<0.001) in comparison to control. Vertical bars on mean values in A represent SE (n = 3). (B) Zymographic detection of POD activity. (C) Western blot analysis of POD.

Aminoguanidine lowers down the specific activity of POD in a concentration-dependent manner and there is a clear cut enhanced impact of aminoguanidine on lowering the enzyme activity (23.6% with respect to control) in salt stressed seedling cotyledons (Fig 6). It is further evident from Western blot analysis that lowering of POD activity due to aminoguanidine treatment is not correlated with the extent of expression of the relevant protein which infact exhibits enhancement in salt-stressed seedling cotyledons as a result of aminoguanidine exposure.

### Salt stressed seedling cotyledons exhibit enhanced tyrosine nitration

**Fig 7:**
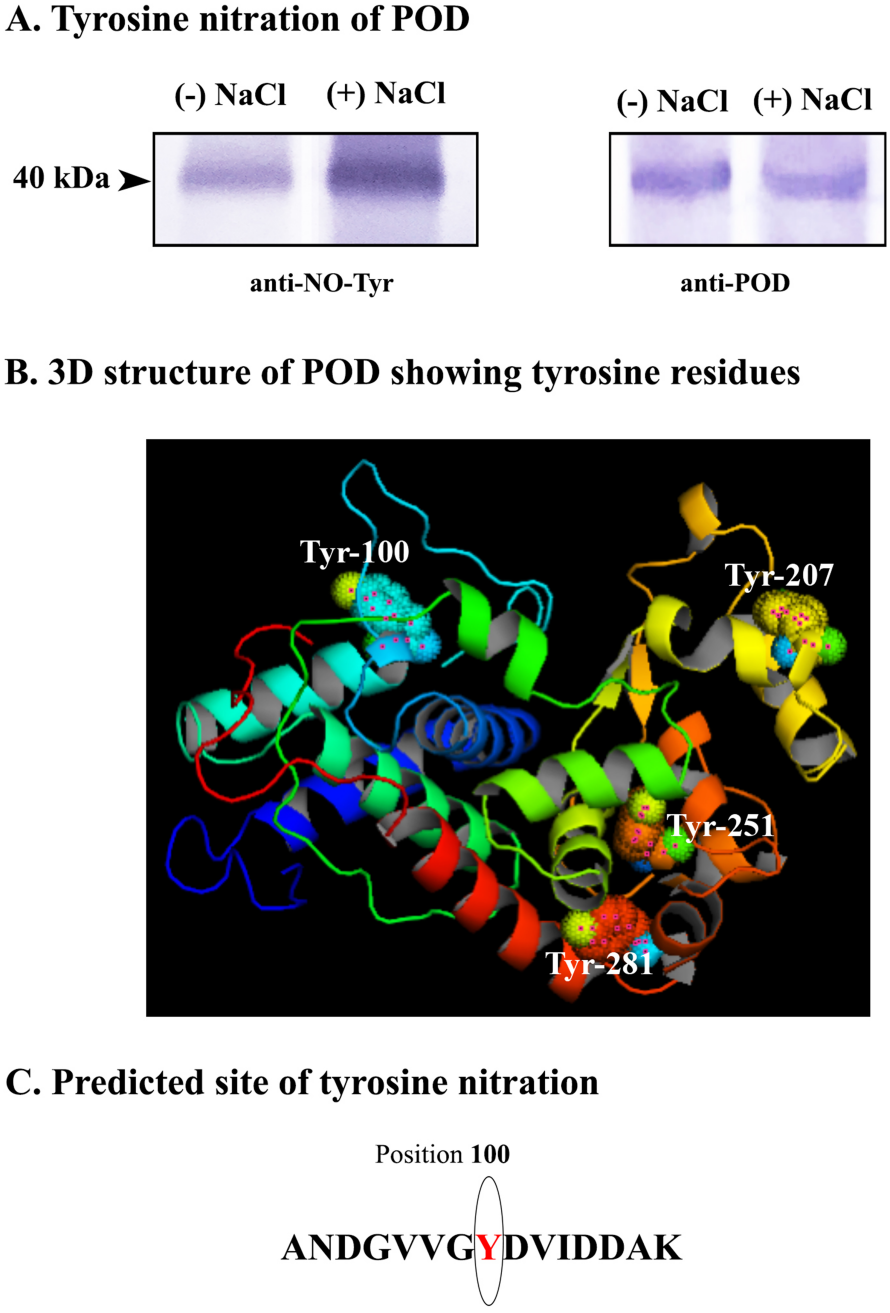
Tyrosine nitration of peroxidase (POD) in sunflower seedling cotyledons. (A) Western blot analysis showing tyrosine nitration of POD using anti-nitrotyrosine antibody. (B) Three-dimensional structure of POD modelled using Swissmodel and PyMol depicting four tyrosine residues at positions 100, 207, 251 and 281. (C) Sites of tyrosine nitration in POD as predicted using GPS-YNO2 1.0.

Whole tissue homogenate (10,000g supernatant) from seedling cotyledons was used for immunoprecipitation of POD using Dynabeads Protein G immunoprecipitation kit (Invitrogen, USA) and co-incubating with anti-POD antibody (P7899, Sigma-Aldrich, USA). The purified POD so obtained, following elution, was analyzed for the extent of tyrosine nitration using anti-nitrotyrosine antibody (AB58411, Sigma-Aldrich, USA). It is evident from Fig 7 that on constant fresh weight basis, cotyledons derived from salt-stressed seedlings exhibited a higher extent of tyrosine nitration of POD as compared to the one from control seedlings.

The amino acid sequence of *Helianthus annuus* L. class III peroxidase was obtained from Peroxibase- the peroxidase database (HaPrx01_QIR8). A three-dimensional structure was designed using SWISS-MODEL and PyMOL to show the four tyrosine residues at positions 100, 207, 251 and 281 (Table 2). Out of the four tyrosine residues found in the amino acid sequence of POD, the one at position 100 was predicted to undergo nitration using GPS-YNO2 1.0 software (Fig 7).

**Table 2:** Tyrosine residues predicted to undergo tyrosine nitration.

Position	Peptide	Score	Cut off	Cluster
100	ANDGVVG**Y**DVIDDAK	1.244	0	Cluster D
207	VRFRGRI**Y**NSPLPID	0.328	0	Cluster A
251	PNTFDNN**Y**FLNLRAS	0.14	0	Cluster C
281	ADSIVTE**Y**ADNPTTF	0.646	0	Cluster E

## Discussion

Although quite a few earlier investigations have provided evidence for salt stress-induced enhanced activity of POD, present work undertakes an intensive and systematic investigation on POD modulation by NaCl stress in sunflower seedling cotyledons as a long-distance impact and differential response of POD activity and POD protein accumulation to salt stress and nitric oxide. Sunflower seedlings grown in the presence of salt (120 mM NaCl) exhibit inhibition of hypocotyl extension and primary root growth, thus leading to growth inhibition (observations from previous works carried out in researcher’s laboratory) [25]. Sodium nitroprusside is an established NO donor and it shows a positive role in growth improvement in control sunflower seedlings up to a concentration of 250 μM (present work). However, its role in overcoming the effects of salt stress is further evident at 500 μM. Results obtained with the use of SNP, however, need to be analyzed with caution keeping in view the fact that SNP also releases cyanide and free iron in solution [29,6]. cPTIO exerts negative impact on seedling growth, it being an established NO scavenger. The mechanism of action of cPTIO involves oxidation of NO to NO_2_, thereby scavenging NO. However, under conditions of excessive NO, cPTIO may facilitate the formation of N_2_O_3_ due to enhanced oxidation of NO thereby highlighting the concentration-dependent duality of cPTIO action [35]. Thus, the results obtained using cPTIO as a NO scavenger need to be analyzed with due caution. Several studies have shown the positive effect of NO on seedling growth and seed germination under different types of abiotic stresses. Exogenous application of SNP significantly enhances seedling growth under salinity stress in rice [24], cotton [36], chamomile [37] and cucumber [38]. These reports, together with our findings on sunflower, suggest that NO is effective in reversing the negative impact of NaCl on seedling growth. Role of nitric oxide in combating NaCl stress in sunflower seedlings observed in the present work is further substantiated by the use of DETA, a more reliable NO donor. NO release by DETA is unaffected by biological reactants. The first order decomposition rate and high stability makes DETA a better source of NO generation [39]. Present observations thus demonstrate that exogenously supplied NO (in the form of DETA or SNP) results in enhanced seedling growth by promoting hypocotyl elongation in sunflower seedlings.

Nitric oxide has been shown to play a critical role in alleviating detrimental effects of abiotic stress in various plants. Under stressful conditions, NO protects the plants from oxidative damage by interacting with the antioxidant enzymes thereby modulating their activities [40]. Additionally, an interplay between NO and sulphur assimilation and also with plant growth regulators such as, auxin, ethylene, cytokinin, helps in abiotic stress tolerance [41, 42]. In *Brassica juncea*, exogenously supplied NO has been reported to protect the plant from inhibitory effects of cadmium stress and salt stress by improving S-assimilation and production of glutathione [26, 28].

The protective role of NO in alleviating oxidative damage by modulating POD activity has been demonstrated in wheat [43] and chickpea [44] under cadmium stress; *Cassia tora* in response to aluminium stress [45]; banana fruits [46] and cucumber seedlings [47] under chilling injury; bean leaves exposed to UV-B radiation [48]; wheat in response to nickel stress [49]; chickpea, tomato, strawberry and cotton in response to salinity stress [50–53]; maize in response to boron-induced oxidative damage [54]; wheat and mung bean leaf discs under high temperature stress [55, 56] and in *Malus* rootstock under drought stress [57]. NO supplementation has also been shown to upregulate the activity of ascorbate peroxidase (APX) in sweet potato (*Ipomoea batata*) coinciding with reduction or prevention of cell death triggered by H_2_O_2_ [58]. Similarly, an increase in the activity of three APX isoforms by NO has been observed in soybean root nodules [59]. In contrast with most of the above-stated earlier reports which show NO-dependent modulation of POD activity using a specific concentration of NO donor, the present work on sunflower highlights concentration dependence of NO impact and it also distinguishes its effect on POD protein abundance and POD activity. Earlier reports have also suggested that cPTIO treatment reverses the positive effects of SNP by lowering the activities of antioxidant enzymes in chickpea [44], roots of *Cassia tora* [45] and sweet potato [58]. The combined observations from the impact of SNP, DETA, cPTIO and aminoguanidine in salt stressed sunflower seedling cotyledons indicate that nitric oxide (endogenous) positively modulates POD activity in seedling cotyledons and the modulation of POD activity by NO is not sensitized by the presence or absence of salt stress.

It is evident from the present series of experiments that NO is probably directly modulating the activity of the active POD enzyme through its binding to specific sites. Various studies have reported that NO can modulate the catalytic activity of peroxidase differently depending on the plant species and severity of stress. NO has been shown to enhance POD activity by promoting the formation of native enzyme from intermediate Compound II in the presence of poor electron donors, such as guaiacol or catechol [60]. Evidence has also been provided to support the possibility of reversible binding of NO to the heme prosthetic group of peroxidase thereby inhibiting xylem POD activity in *Zinnia elegans*. NO has been suggested to bind to the inactive form of peroxidase [61], thus withdrawing it from the catalytic cycle. It has also been speculated that NO directly interacts with the iron atom in the heme moiety of POD, forming an iron-nitroxy complex, thus reversibly inhibiting enzyme activity in tobacco [62].

The present study reveals that a probable NO-POD crosstalk exists in sunflower seedling cotyledons. NO positively modulates POD activity but the mechanism by which NO regulates POD activity remains to be investigated. Ascorbate peroxidase (APX) activity in plants has already been shown to be influenced by various NO-mediated post-translational modifications. Tyrosine nitration of cytoplasmic APX has been reported in *Arabidopsis* [63]. In citrus plants subjected to salt stress, cytoplasmic APX has been shown to get nitrated in roots [64]. S-nitrosylation of APX has been reported as well. S-nitrosylation of cytosolic ascorbate peroxidase (APX 1) at Cys32 residue leads to enhanced enzyme activity under conditions of oxidative stress [65]. Additionally, a rapid decrease in cytosolic APX activity due to S-nitrosylation has also been observed in tobacco Bright Yellow-2 cells undergoing programmed cell death [66]. Evidence for dual regulation of cytosolic APX has also been recently provided in pea showing that S-nitrosylation at Cys 32 residue enhances APX activity in pea, while nitration at Tyr5 and Tyr235 residues by peroxynitrite leads to activity inhibition [67]. Present investigations demonstrate enhanced tyrosine nitration of POD as a mechanism to increase enzyme activity accompanying salt stress. Nevertheless, POD under investigation in the present work does indicate that its nitration (and consequent effect on POD activity) is modulated differently under control and salt-stressed conditions.

To sum up, present work correlates NO-POD interaction as a rapid long distance signaling response which operates irrespective of salt stress. It demonstrates NO-dependent modulation of POD protein abundance and activity. Role of NO in combating NaCl stress in sunflower seedlings observed in the present work is further substantiated by the use of DETA (in addition to SNP), a more reliable NO donor. Peroxidases are heme-containing enzymes and are, therefore, one of the potential targets of NO action. This work further highlights concentration-dependence of the impact of NO and distinguishes its effect on POD protein abundance and specific activity of POD. The combined observations from the impact of SNP, DETA, cPTIO and aminoguanidine in salt stressed seedling cotyledons indicate that nitric oxide (endogenous) positively modulates POD activity and this NO-POD crosstalk is not sensitized by salt stress. Finally, present investigations demonstrate enhanced tyrosine nitration of POD as a probable mechanism to increase enzyme activity accompanying salt stress.

## Supporting information

S1 Dataset Dataset for POD activity in the presence of NO donors and scavenger. (XLSX)

## Abbreviations

APX: Ascorbate peroxidase
CAT: Catalase
cPTIO: 2-(4-carboxyphenyl)-4,4,5,5-tetramethylimidazoline-1-oxyl-3-oxide
DETA: Diethylenetriamine NONOate
GPX: Glutathione peroxidase
NO: Nitric oxide
POD: Peroxidase
PRX: Peroxiredoxin
ROS: Reactive oxygen species
SNP: Sodium nitroprusside
